# Butyrate Is Associated with the Antidepressant Effects of *Weizmannia coagulans* BC99: Functional Similarity of a Microbial Metabolite in the Microbiota–Gut–Brain Axis

**DOI:** 10.3390/ijms27094082

**Published:** 2026-05-02

**Authors:** Yiqing Zhou, Yuwan Li, Shanshan Tie, Yao Dong, Shuguang Fang, Ying Wu, Shaobin Gu

**Affiliations:** 1 College of Food and Bioengineering, Henan University of Science and Technology, Luoyang 471000, China; zhouyiqing1999@163.com (Y.Z.); liyuwan91@163.com (Y.L.); tieshanshan@haust.edu.cn (S.T.); yingwu@haust.edu.cn (Y.W.); 2 Henan Provincial Key University-Enterprise Joint R&D Center of Probiotics Scientific Evidence-Based Research and Industrial Application, Luoyang 471023, China; yao.dong@wecare-bio.com (Y.D.); frank.fang@wecare-bio.com (S.F.); 3 Henan Engineering Research Center of Food Microbiology, Luoyang 471000, China

**Keywords:** depression, gut-brain axis, psychobiotics, butyrate, short-chain fatty acids, neuroinflammation, BDNF/TrkB/CREB, probiotics

## Abstract

Butyrate, a short-chain fatty acid derived from the gut microbiota, has been linked to depression through correlational studies; however, whether it might act as a sufficient downstream mediator of the antidepressant effects of a probiotic remains poorly understood. To explore this, a chronic unpredictable mild stress (CUMS) rat model was established to evaluate the potential antidepressant effects of *Weizmannia coagulans* BC99. Behavioral assessments included the sucrose preference test (SPT), forced swim test (FST), tail suspension test (TST), and open field test (OFT). In addition, 16S rRNA sequencing, serum metabolomics, and short-chain fatty acid (SCFA) profiling were performed. Levels of inflammatory cytokines (IL-1β, IL-6, IL-4, and LPS) and brain-derived neurotrophic factor (BDNF) were measured in serum, hippocampus, and colon by ELISA. An independent sodium butyrate supplementation experiment was conducted to test functional sufficiency, and hippocampal BDNF/TrkB/CREB signaling was assessed by Western blotting. Treatment with BC99 was associated with alleviation of CUMS-induced depressive-like behaviors, increased butyrate levels, reduced neuroinflammation (IL-1β, IL-6, LPS, and IL-4), and restored hippocampal BDNF levels. BC99 also enriched butyrate-producing bacterial taxa (e.g., *Lactobacillus*, *Bifidobacterium*, *Faecalibaculum*) and normalized tryptophan and sphingolipid metabolism. Notably, sodium butyrate alone recapitulated several of the behavioral and anti-inflammatory effects observed with BC99 and, as shown by Western blot, partially restored hippocampal BDNF/TrkB/CREB signaling, which was impaired in CUMS rats. Together, these findings suggest that butyrate may be associated with the antidepressant effects of *W. coagulans* BC99, potentially acting through suppression of neuroinflammation and activation of the BDNF pathway. Our results support further investigation of butyrate-enhancing strategies as a nutritional approach for depression.

## 1. Introduction

Depression affects over 280 million people globally; however, current pharmacological treatments are often limited by inadequate efficacy and undesirable side effects [1,2]. The gut microbiota has emerged as an important regulator of brain function through the microbiota–gut–brain axis, with microbial metabolites—particularly short-chain fatty acids (SCFAs)—being proposed as key signaling molecules [3,4,5,6].

Among SCFAs, butyrate has garnered increasing attention for its neuroprotective properties [7,8,9]. Butyrate serves as the primary energy source for colonocytes, enhances gut barrier integrity, inhibits NF-κB-mediated neuroinflammation [10,11], and can cross the blood–brain barrier to modulate brain-derived neurotrophic factor (BDNF) expression [12,13]. Clinical studies have reported reduced butyrate levels in patients with major depressive disorder [14], while animal studies suggest that butyrate supplementation may ameliorate depressive-like behaviors [15,16,17,18]. A recent 2026 review highlighted that butyrate deficiency in depression may stem from a lack of butyrate-producing bacteria such as *Eubacterium* and *Faecalibacterium* [19]. Together, these observations suggest that butyrate may represent a promising therapeutic target for depression.

Probiotics that enhance butyrate production have therefore been proposed as potential psychobiotics [20]. However, whether butyrate is merely associated with the antidepressant effects of probiotics or plays a functional role remains an open question. While numerous studies have reported correlations between probiotic-induced microbiota changes, increased butyrate levels, and improved behavioral outcomes [21,22,23,24,25], direct evidence demonstrating that butyrate alone can recapitulate probiotic effects is limited. Addressing this gap may be important for mechanistic understanding and therapeutic development: if butyrate is functionally relevant, then butyrogenic probiotics or direct butyrate supplementation could represent a targeted strategy.

*Weizmannia coagulans* (formerly *Bacillus coagulans*) is a spore-forming, lactic acid-producing bacterium that is Generally Recognized as Safe (GRAS) [26,27]. Previous studies have suggested that it can modulate gut microbiota and alleviate metabolic disorders [28,29,30,31,32]; however, whether *W. coagulans* exerts antidepressant effects through butyrate-related mechanisms has not been investigated.

To address this gap, the present study used a chronic unpredictable mild stress (CUMS) rat model to: (i) evaluate whether *W. coagulans* BC99 alleviates depressive-like behaviors and increases butyrate production; (ii) characterize BC99-induced changes in gut microbiota and metabolic profiles; (iii) assess the involvement of BDNF/TrkB/CREB signaling; and (iv) explore the functional role of butyrate through separate sodium butyrate supplementation. By directly comparing the effects of BC99 and butyrate, our findings suggest that butyrate may serve as a downstream mediator of antidepressant effects, potentially contributing to a causal understanding of the relationship between microbial metabolite production and host neuroimmune benefits.

## 2. Results

### 2.1. BC99 Alleviates Depressive-like Behaviors and Increases Butyrate Levels

**Figure 1:**
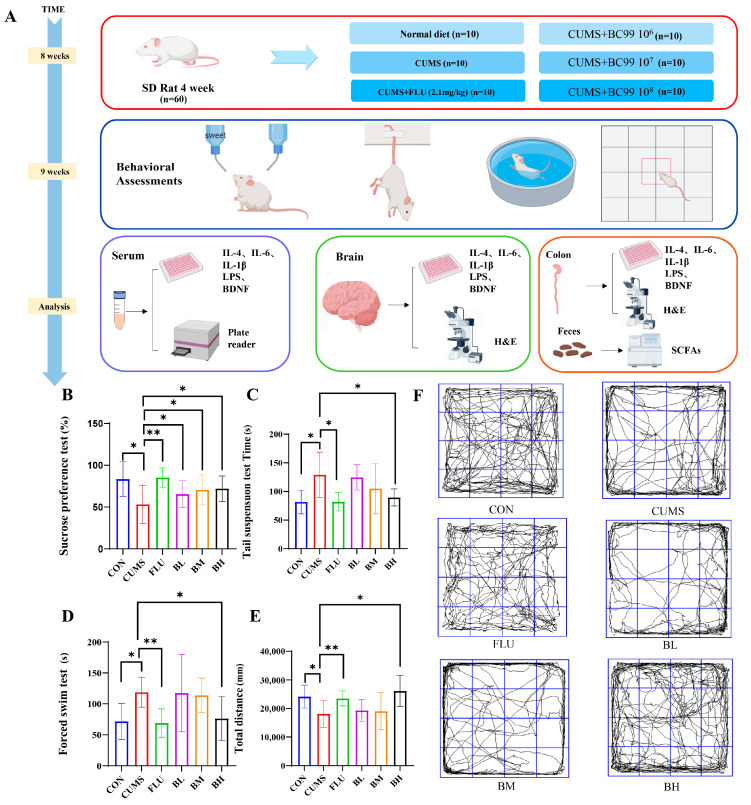
BC99 alleviates depressive-like behaviors in CUMS rats. (**A**) Experimental design and workflow. (**B**) Sucrose preference (%) in the sucrose preference test. (**C**) Immobility time in the tail suspension test. (**D**) Immobility time in the forced swim test. (**E**) Total distance traveled in the open field test. (**F**) Trajectory diagram of rat’s activity in the OFT. Data are presented as mean ± standard deviation (SD) (*n* = 6). Light blue represents the control group, red represents the model group, green represents the positive drug group, purple represents the BC99 low-dose group, yellow represents the BC99 medium-dose group, and black represents the BC99 high-dose group. * *p* < 0.05, ** *p* < 0.01 vs. the CUMS group.

To evaluate the effects of BC99 on depression, we established a CUMS rat model (Figure 1A). The sucrose preference test, tail suspension test, open field test, and forced swimming test were used to assess depressive-like behaviors. As shown in Figure 1B, rats subjected to 8 weeks of CUMS showed a decrease in sucrose preference, indicating anhedonia. Following CUMS exposure, changes in mobility and immobility time were observed in the forced swimming test, tail suspension test, and open field test (Figure 1C–F). After fluoxetine treatment, the treated rats displayed an increased pleasure response and improved immobility time. With BC99 supplementation, rats in the BC99 group appeared to be more active and healthier compared to the model group, particularly in the BH group. Relative to the CUMS group, BC99 administration was associated with increased sucrose preference. Additionally, immobility times in the tail suspension and forced swimming tests, as well as total travel distance in the open field test, were improved in the BC99 group relative to the CUMS group.

**Figure 2:**
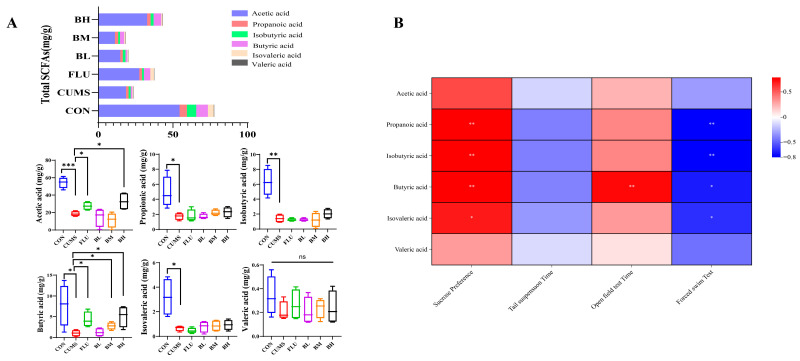
Modulatory effects of BC99 on SCFAs in CUMS-induced depressive rats. (**A**) Contents of SCFAs. (**B**) Correlation heatmap between SCFAs and behavior test. Data are presented as mean ± standard deviation (SD) (*n* = 6). Light blue represents the control group, red represents the model group, green represents the positive drug group, purple represents the BC99 low-dose group, yellow represents the BC99 medium-dose group, and black represents the BC99 high-dose group. * *p* < 0.05, ** *p* < 0.01, *** *p* < 0.001 vs. the CUMS group.

Fecal SCFA analysis suggested that BC99 supplementation was associated with increased butyrate levels compared with the CUMS group (Figure 2A). Acetate and propionate showed similar trends but did not reach statistical significance. Butyrate levels were positively correlated with sucrose preference and OFT and negatively correlated with immobility time in the FST (Figure 2B), suggesting an association between butyrate production and behavioral improvement.

### 2.2. BC99 Suppresses Neuroinflammation and Restores Hippocampal BDNF Levels

**Figure 3:**
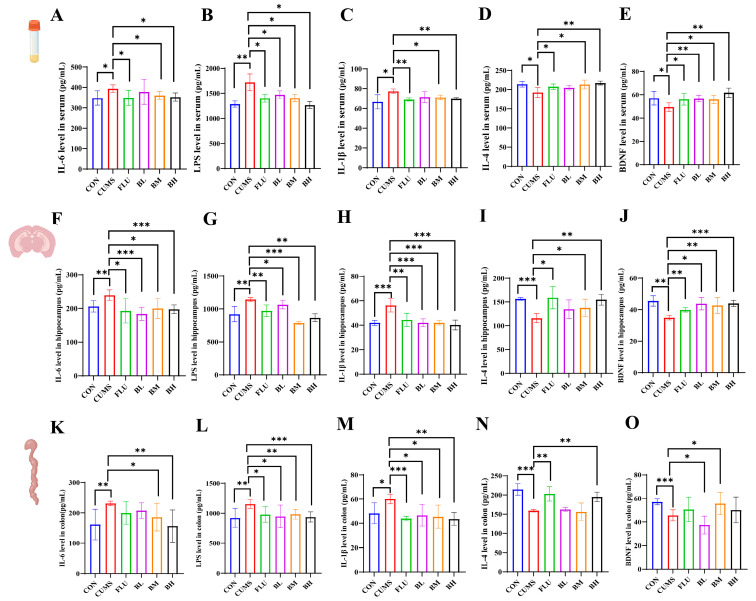
Effects of BC99 on cellular structures, neurotransmitters, and inflammatory markers in serum, hippocampus, and colon. (**A**–**E**) Serum levels of IL-6, LPS, IL-1β, IL-4, and BDNF. (**F**–**J**) Levels of IL-6, IL-4, IL-1β, LPS, and BDNF in hippocampal tissue. (**K**–**O**) Levels of IL-6, IL-4, IL-1β, LPS, and BDNF in colonic tissue. Data are presented as mean ± standard deviation (SD) (*n* = 6). Light blue represents the control group, red represents the model group, green represents the positive drug group, purple represents the BC99 low-dose group, yellow represents the BC99 medium-dose group, and black represents the BC99 high-dose group. * *p* < 0.05, ** *p* < 0.01, *** *p* < 0.001 vs. the CUMS group.

Depression has been linked to inflammatory responses. We analyzed inflammatory cytokine levels in serum, hippocampus, and colon samples. The CUMS group showed elevated levels of IL-6, LPS, and IL-1β in serum compared to the CON, FLU and BC99 groups (Figure 3A–C). In contrast, BC99-treated rats showed higher levels of the anti-inflammatory cytokine IL-4 and the neurotrophic factor BDNF (Figure 3D,E).

H&E staining of the hippocampal CA1 region suggested that the CUMS group had looser neuronal arrangement, some degree of degeneration, and deeply stained cell bodies compared to the CON group (Figure S1). BC99 intervention was associated with improved neuronal morphology, with more compact and orderly arrangements, as well as reduced edema and degeneration, particularly in the BH group. Hippocampal levels of IL-1β, LPS, and IL-6 were higher in the CUMS group, whereas IL-4 and BDNF were reduced (Figure 3F–J). BC99 intervention tended to reduce pro-inflammatory markers and increased IL-4 and BDNF levels towards those observed in the CON group.

Colonic H&E staining indicated villous atrophy, epithelial cell shedding, and submucosal separation in the CUMS group (Figure S2). BC99 treatment appeared to alleviate these pathological changes, with the most noticeable effects observed in the BM group. Colonic levels of IL-1β, IL-6, and LPS were higher in the CUMS group and reduced after BC99 treatment, whereas IL-4 and BDNF levels were higher (Figure 3K–O).

### 2.3. BC99 Enriches Butyrate-Producing Bacterial Taxa

**Figure 4:**
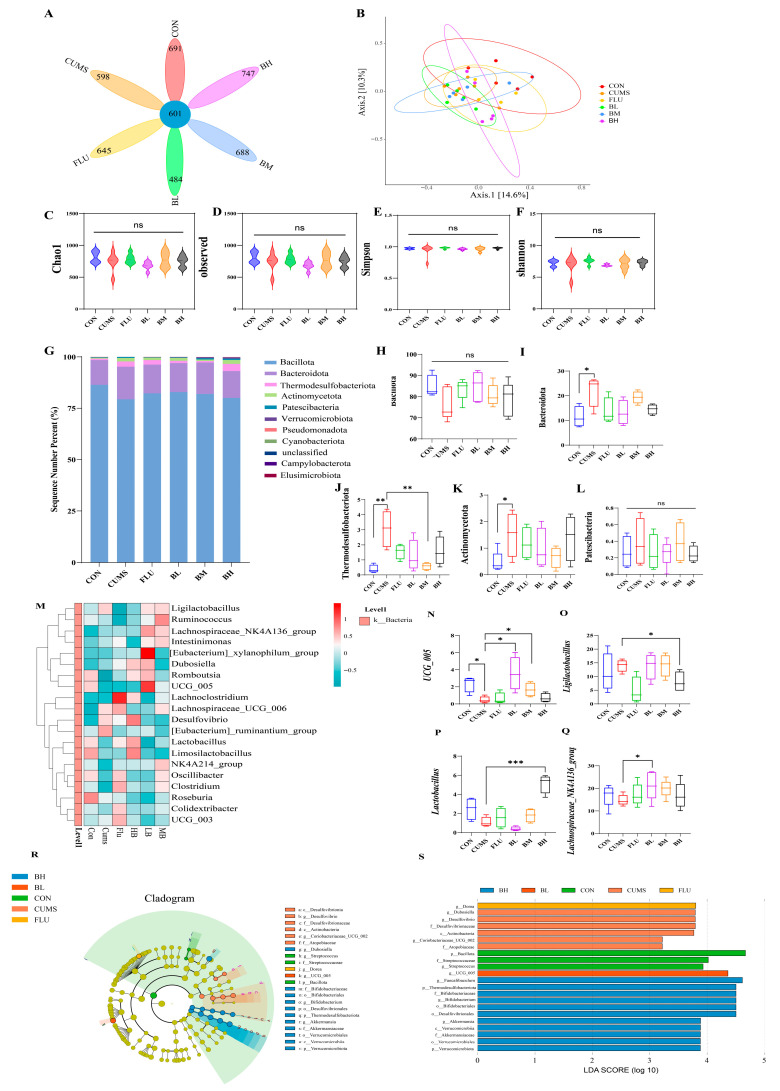
Regulatory effects of BC99 on gut bacterial microstructure and composition in CUMS rats. (**A**) The number in the center of the Venn diagram of OTUs represents the total OTUs shared by all six groups, and the number on each petal represents the OTUs unique to that group. (**B**) PCoA plot illustrates differences in microbial community structure among groups. (**C**–**F**) Violin plot of alpha diversity illustrating the diversity of each group. Taxonomic annotation was performed from phylum to genus level. (**G**–**L**) The phylum level relative abundance histogram illustrates the relative abundance and composition of the major phyla in each group. (**M**–**Q**) The relative abundance histogram at genus level illustrates the relative abundance and composition of key bacterial genera in each group. (**R**) A cladogram illustrating the taxa significantly enriched in each group at the phylum to genus level. (**S**) The histogram of LDA distribution illustrates the differential species with LDA score > 4.0. Data are presented as mean ± standard deviation (SD) (*n* = 6). Light blue represents the control group, red represents the model group, green represents the positive drug group, purple represents the BC99 low-dose group, yellow represents the BC99 medium-dose group, and black represents the BC99 high-dose group. * *p* < 0.05, ** *p* < 0.01, *** *p* < 0.001 vs. the CUMS group.

To investigate BC99-induced changes in the gut microbiota, we performed 16S rRNA sequencing on cecal contents. The control group had 691 unique operational taxonomic units (OTUs), the CUMS group had 598 unique OTUs, the FLU group had 645 unique OTUs, and the BC99 low-, medium-, and high-dose groups had 484, 688, and 747 unique OTUs, respectively. There were 601 OTUs shared among all groups (Figure 4A). Principal coordinates analysis (PCoA) suggested that the CON group clustered closer to the FLU group, while the BH group appeared to shift toward the CON group (Figure 4B), indicating that BC99 intervention may have partially restored the microbiota composition. While α-diversity did not differ significantly among groups (Figure 4C–F), community composition (β-diversity) and relative taxon abundance appeared to be altered. Although alpha diversity did not differ significantly among groups, indicating no drastic changes in species richness or evenness, beta diversity analysis using PCoA revealed that the gut microbial community structure of the BC99-treated group shifted significantly toward that of the normal control group. These results suggest that CUMS and BC99 intervention primarily affect microbial community composition and the relative abundance of specific taxa rather than overall species richness. This further highlights the potential importance of specific functional bacterial populations modulated by BC99 intervention in mediating antidepressant effects. Taxonomic annotation of OTUs was performed from phylum to genus level. The relative abundances of microbial communities were analyzed at both the phylum level and the genus level. At the phylum level, the proportions of Verrucomicrobia and Bacteroidetes decreased in BC99 groups, while Firmicutes, Proteobacteria, and Actinobacteria increased (Figure 4G–L).

At the genus level, the CUMS group showed higher abundances of *Desulfovibrio*, *[Eubacterium]_ruminantium_group*, and *Lachnospiraceae_UCG_006*, and reduced levels of beneficial bacteria such as *Lachnospiraceae_NK4A136_group*, *UCG_005*, *Lachnoclostridium*, and *Lactobacillus* (Figure 4M–Q). *Lachnospiraceae_UCG_006* was also enriched in the FLU group. BC99 supplementation reversed these changes. LEfSe analysis indicated enrichment of beneficial bacteria including *Lactobacillus*, *Akkermansia*, *Bifidobacterium*, *Faecalibaculum* and *Bifidobacteriales* in the BH group (Figure 4R,S). *Faecalibaculum* has been shown to be closely associated with butyrate levels [33,34].

Correlation analysis revealed positive correlations between the abundance of these taxa and butyrate levels. BDNF levels were positively correlated with butyrate concentrations, suggesting a potential link between butyrate and BDNF restoration (Figure S3).

### 2.4. BC99 Normalizes Serum Metabolic Profiles Related to Tryptophan and Sphingolipid Metabolism

**Figure 5:**
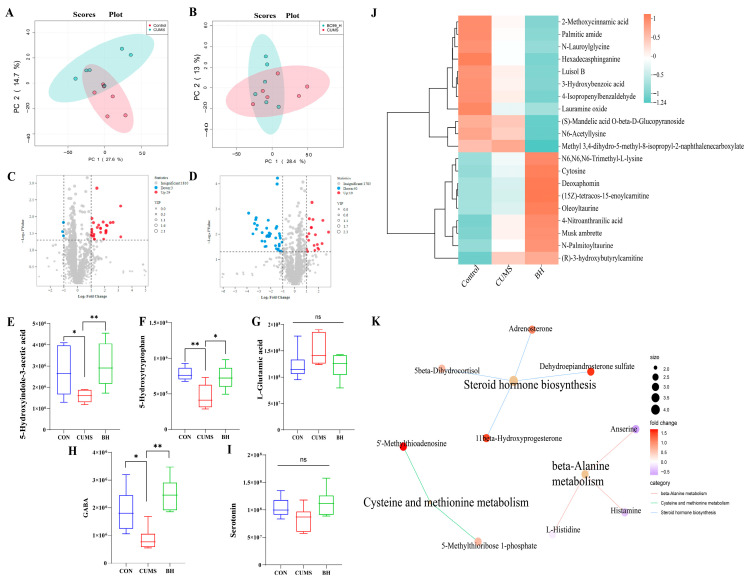
(**A**) Principal component analysis (PCA) score plot of the CON and CUMS groups. (**B**) PCA score plot of the CUMS and BC99 groups. (**C**) Volcano plot (CON/CUMS). (**D**) Volcano plot (CUMS/BC99). (**E**–**I**) Levels of 5-Hydroxyindole-3-acetic acid, 5-Hydroxytryptophan, GABA, Serotonin, and L-Glutamic acid among the CON, CUMS, and BC99 groups. (**J**) Cluster heatmap of metabolites in the CON, CUMS, and BC99 groups. (**K**) KEGG enrichment analysis of differential metabolites across different groups. Data are presented as mean ± standard deviation (SD) (*n* = 6). Light blue represents the control group, red represents the model group, green represents the positive drug group, purple represents the BC99 low-dose group, yellow represents the BC99 medium-dose group, and black represents the BC99 high-dose group. * *p* < 0.05, ** *p* < 0.01 vs. the CUMS group.

Targeted serum metabolomics was used to investigate the impact of BC99. Using UHPLC-MS/MS analysis, we detected approximately 1862 metabolite features across all serum samples. Principal component analysis (PCA) showed separation between the CON and CUMS groups, as well as between the CUMS and BC99 groups (Figure 5A,B), suggesting that BC99 intervention was associated with alterations in the metabolites profile of CUMS rats. Furthermore, *t*-tests were used to identify differentially accumulated metabolites between groups. As shown in Figure 5C,D, there were a total of 32 differentially accumulated metabolites between the CUMS and CON groups, of which 29 were up-regulated and 3 were down-regulated. Between the BC99 and CUMS groups, 59 metabolites were identified, with 19 up-regulated and 40 down-regulated. Differential metabolites were defined based on *p* < 0.05 and a fold change (FC) > 1.5 or <0.67 (1/1.5) for up-regulated or down-regulated metabolites, respectively.

Compared with the CON group, the CUMS group showed alterations in several key metabolites. 5-Hydroxyindole-3-acetic acid, 5-Hydroxytryptophan (5-HTP), and GABA were lower, whereas the excitatory neurotransmitter glutamate was higher (Figure 5E–I). Serum serotonin itself did not show notable changes. Relative to the CON group, the CUMS group also exhibited lower levers of the sphingolipid hexosylsphingosine and (R)-3-hydroxybutylcarnitine. Neuro-modulatory metabolites such as oleoyltaurine and N-Palmitoyltaurine also showed decreasing trends (Figure 5J). BC99 intervention was associated with improvements in these disturbances, appearing to reverse (R)-3-hydroxybutyrylcarnitine levels and up-regulating oleoyltaurine and N-Palmitoyltaurine. KEGG enrichment analysis indicated that differential metabolites were primarily involved in steroid hormone biosynthesis, beta-alanine metabolism, and cysteine and methionine metabolism (Figure 5K). Importantly, correlation analysis revealed that serotonin levels were significantly positively correlated with IL-4 and BDNF, significantly negatively correlated with IL-6, IL-1β, and LPS, and significantly positively correlated with sucrose preference (Figure S4). These metabolic changes may reflect the multi-target regulation of the gut–brain axis by butyrate, although further studies are needed to establish direct causal links.

### 2.5. Butyrate Supplementation Alone Recapitulates BC99’s Antidepressant and Anti-Inflammatory Effects and Activates BDNF/TrkB/CREB Signaling

We identified *Lactobacillus*, *Bifidobacterium* and *Faecalibaculum* as bacteria associated with butyrate levels [35,36]. Our analysis suggested that butyrate may be a key metabolite, as it was positively correlated with beneficial microorganisms, and negatively correlated with inflammatory markers and depressive-like behaviors. To explore its functional role, we supplemented CUMS rats with sodium butyrate (30 mg/kg) [37]. This dose was selected based on previous studies showing efficacy in CUMS models and to mimic the physiological increase in butyrate observed after BC99 intervention.

**Figure 6:**
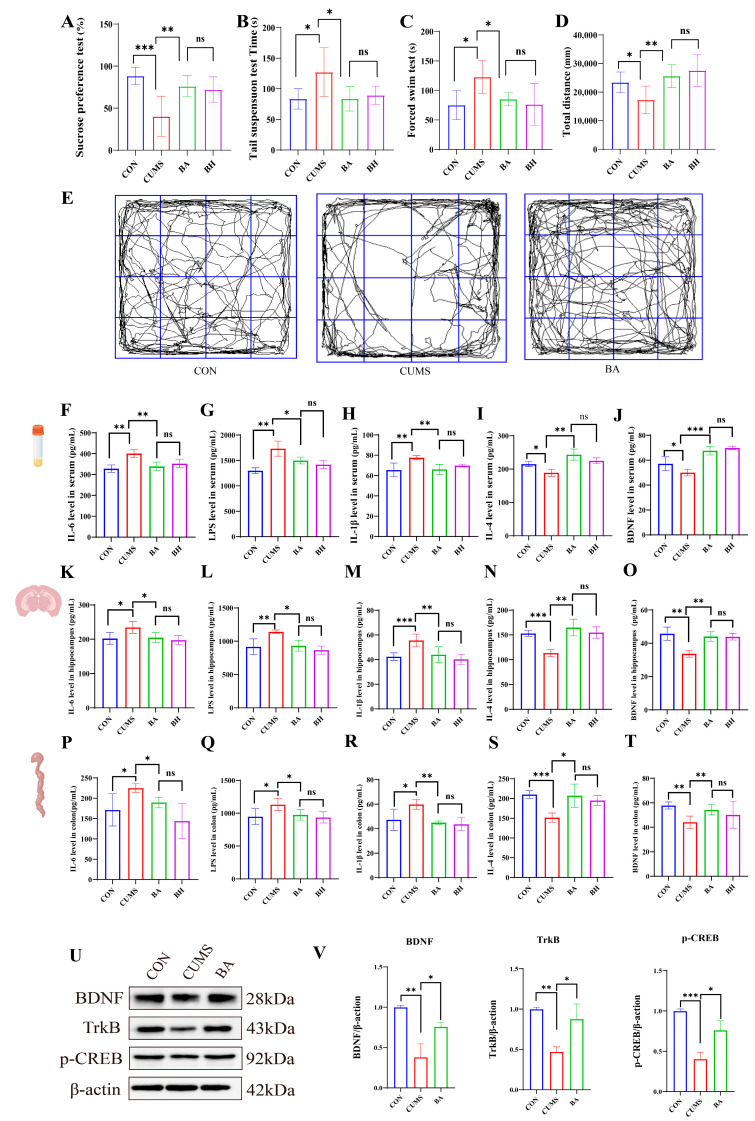
BA can alleviate depression-like behaviors in CUMS rats, reduce inflammation, and improve tissue structure. (**A**) Sucrose preference (%) in the sucrose preference test. (**B**) Immobility time in the tail suspension test. (**C**) Immobility time in the forced swim test. (**D**) Total distance traveled in the open field test. (**E**) Trajectory diagram of rat’s activity in the OFT. (**F**–**J**) Serum levels of IL-6, LPS, IL-1β, IL-4, and BDNF. (**K**–**O**) Levels of IL-6, IL-4, IL-1β, LPS, and BDNF in hippocampal tissue. (**P**–**T**) Levels of IL-6, IL-4, IL-1β, LPS, and BDNF in colonic tissue. (**U**) Band diagram of BDNF, TRKB, p-CREB, β-actin proteins in hippocampal tissue. (**V**) Protein expression of BDNF, TRKB, p-CREB in hippocampal tissue. Data are presented as mean ± standard deviation (SD) (*n* = 6). Light blue represents the control group, red represents the model group, green represents the positive drug group, purple represents the BC99 low-dose group, yellow represents the BC99 medium-dose group, and black represents the BC99 high-dose group. * *p* < 0.05, ** *p* < 0.01, *** *p* < 0.001 vs. the CUMS group.

Butyrate treatment was associated with improvements in the behavioral outcomes assessed, including increasing sucrose preference, reducing immobility in FST and TST, and restoring locomotor activity in OFT (Figure 6A–E). These effects showed no statistically significant differences compared with the high-dose BC99 group.

Butyrate also appeared to exhibit anti-inflammatory effects similar to those of BC99: in serum, butyrate reduced IL-6, LPS, and IL-1β while increasing IL-4 and BDNF (Figure 6F–J); in the hippocampus, butyrate reduced IL-6, LPS, and IL-1β while increasing IL-4 and BDNF (Figure 6K–O); and in the colon, butyrate reduced IL-6, LPS, and IL-1β while increasing IL-4 and BDNF (Figure 6P–T). H&E staining suggested that butyrate was associated with preservation of hippocampal neuronal architecture and colonic mucosal integrity (Figure S5).

It is worth noting, Western blot analysis of hippocampal tissue from the butyrate validation experiment indicated that CUMS was associated with reduced phosphorylation of TrkB and CREB, as well as lower total BDNF protein levels. Sodium butyrate treatment alone partially restored TrkB, p-CREB and BDNF expression to levels comparable to those control rats (Figure 6U,V). These results are consistent with the hypothesis that butyrate may contribute to activating the BDNF/TrkB/CREB signaling cascade, potentially linking butyrate to hippocampal neuroplasticity. Notably, no statistically significant differences were detected between the butyrate group and the high-dose BC99 group in all behavioral indicators and key inflammatory factors, suggesting that supplementation with butyrate alone can effectively mimic the overall effects produced by high-dose BC99 intervention. Following butyrate intervention, the hippocampal tissue disorganization induced by CUMS appeared to improve, and colonic inflammation appeared to be reduced (Figure S6). These results suggest that sodium butyrate alone may phenocopy several behavioral and anti-inflammatory effects of BC99, indicating functional similarity and supporting its potential role as a downstream effector molecule in the mechanism of BC99.

## 3. Discussion

The present study offers evidence consistent with the hypothesis that butyrate may serve as a functional mediator of some of the antidepressant effects of a probiotic, suggesting that butyrate could act as a potential postbiotic metabolite in gut–brain communication. Using a CUMS rat model, we observed that *Weizmannia coagulans* BC99 alleviates depressive-like behaviors, suppresses neuroinflammation, and activates BDNF/TrkB/CREB signaling effects that were also observed with independent sodium butyrate supplementation. These findings contribute to addressing a gap in the field: although numerous studies have reported correlations between probiotic-induced microbiota changes and improved mental health outcomes [38,39,40], direct evidence demonstrating the functional role of a single microbial metabolite remains limited. Our results suggest that butyrate may be more than a correlational biomarker and could act as a downstream mediator, which may have implications for the development of next-generation psychobiotics and postbiotic-based interventions for depression and stress-related disorders.

Mechanistically, BC99 was associated with enrichment of butyrate-producing bacterial taxa (*Lactobacillus*, *Bifidobacterium*, *Faecalibaculum*), which were positively correlated with butyrate levels. Among the bacterial genera enriched by BC99, *Faecalibaculum* is particularly interesting. Recent studies have clearly identified *Faecalibaculum rodentium* as a butyrate-producing genus in the gut, whose abundance strongly correlates with fecal butyrate levels [41]. The enrichment of *Faecalibaculum* by BC99 observed in this study provides microbiological evidence consistent with a role for BC99 in elevating butyrate levels. This finding is consistent with recent evidence that depression is characterized by depletion of SCFA-producing bacteria and reduced microbial diversity [42,43,44]. The functional properties of this psychobiotic candidate strain may extend beyond butyrate production to encompass metabolic restoration [29,45]. Notably, beyond butyrate production, BC99—as a spore-forming bacterium—may exhibit enhanced gastrointestinal survivability. Additionally, L-lactate generated during BC99 fermentation could influence the central nervous system independently of butyrate, potentially via monocarboxylate transporters, or could serve as a cross-feeding substrate for other butyrate-producing bacteria. Therefore, the antidepressant effects of BC99 are likely multi-targeted, with butyrate being a key but not the sole mediator. Serum metabolomics suggested that BC99 was associated with normalization of tryptophan metabolism intermediates (5-HTP, 5-HIAA) and sphingolipid profiles, indicating a possible increase in serotonin biosynthesis. These findings resonate with the growing understanding that gut bacteria can influence neurotransmitter systems including serotonin, GABA, and dopamine pathways—through multiple mechanisms [46,47,48,49]. Furthermore, our observation that BC99 was associated with lower levels of pro-inflammatory cytokines (IL-1β, IL-6) and LPS, and with restoration of IL-4, suggests a role in modulating neuroinflammation, a key pathway linking gut dysbiosis to brain dysfunction and cognitive decline [50,51]. The butyrate validation experiment, which recapitulated several of these effects and partially activated BDNF/TrkB/CREB signaling, is consistent with the possibility that this microbial metabolite may influence neurogenesis and blood–brain barrier function [52,53], as previously suggested by HDAC inhibition mechanisms [54].

The translational implications of our findings may warrant further investigation in the context of emerging microbiome-based therapies for mental health. Current evidence suggests that lifestyle factors-including diet and chronic stress profoundly influence gut microbiota composition and, consequently, brain health [55,56,57]. Beyond butyrate, BC99 normalized multiple metabolic pathways implicated in depression, including sphingolipid metabolism, steroid hormone biosynthesis, and amino acid metabolism, suggesting broader metabolic restoration that could contribute to the antidepressant phenotype. Changes in serum metabolites may represent secondary consequences of butyrate-mediated improvements in intestinal barrier function and reductions in systemic inflammation. However, establishing a direct causal link between these metabolic changes and the antidepressant-like phenotype will require future experimental validation. Approaches such as metabolite supplementation or inhibition of synthetic enzymes are needed to address this question. Several limitations of the present study should be acknowledged. First, the sample size of six rats per group is relatively small, which limits the statistical power and generalizability of our findings; larger cohorts are needed to confirm these results. Second, the gut microbiota of butyrate-supplemented rats was not analyzed; therefore, we cannot exclude the possibility that exogenous butyrate exerts secondary effects on microbial community structure that contribute to its benefits. Third, although the correlation between BC99-induced butyrate elevation and BDNF restoration suggests a link, this study does not directly demonstrate that butyrate is necessary for BC99 to exert its antidepressant effects. To help establish a causal relationship, future experiments, such as those using butyrate synthase knockout strains, GPR41/43-specific antagonists, or HDAC inhibitors, could be considered to assess the necessity of butyrate. Direct evidence that BC99 activates the BDNF/TrkB/CREB pathway via butyrate awaits future studies incorporating specific inhibitors or knockdown models. Fourth, this study was conducted only in male Sprague-Dawley rats, and potential sex differences were not assessed. Given the higher prevalence of depression in females and the existence of sex-dimorphic pathophysiological mechanisms, future studies should validate our findings in both sexes. Fifth, although sodium butyrate was administered by gavage, we cannot completely rule out the possibility that its sour taste or associated gastrointestinal discomfort influenced behavioral outcomes via vagal pathways. Future studies should employ blinded designs and include taste controls to eliminate this confounding factor. Sixth, a recent randomized, double-blind, placebo-controlled clinical trial provided preliminary evidence supporting the clinical translation of *W. coagulans* BC99 [58]. The study reported that in subjects with anxiety and depressive symptoms, eight weeks of BC99 supplementation (5 × 10^9^ CFU/day) improved HAMD and HAMA scores, increased fecal short-chain fatty acid (SCFA) levels and neurotransmitters such as GABA, and also enriched the beneficial bacterium *Faecalibacterium*. These clinical observations align with our findings in the CUMS animal model, where BC99 ameliorated depressive-like behaviors, increased butyrate levels, and modulated neuroinflammation, further supporting the potential of BC99 as a candidate psychobiotic. Translation to humans requires clinical validation in depressed patients. Future studies should investigate the specific butyrate receptors and signaling pathways mediating its antidepressant effects, whether butyrate and other SCFAs act synergistically, and the optimal dosing and delivery strategies for clinical application.

## 4. Materials and Methods

### 4.1. Materials and Reagents

The *W. coagulans* BC99 (hereinafter referred to as BC99) strain was obtained from Wecare Probiotics Co., Ltd. (Suzhou, China). The BC99 bacterial powder contains 1 × 10^11^ CFU/g. Appropriate doses of bacterial suspension were prepared by diluting in sterile saline. Once prepared, it was stored as a backup ingredient at 4 °C. Fluoxetine was purchased from Suzhou Sinopharm Pharmaceutical Industry Co., Ltd. (Suzhou, China). ELISA kits for rat interleukin-4 (IL-4), interleukin-6 (IL-6), interleukin-1β (IL-1β), lipopolysaccharide (LPS), and brain-derived neurotrophic factor (BDNF) were sourced from Shanghai Hepeng Company (Shanghai, China). BDNF, TrkB, and p-CREB, β-actin antibodies were purchased from Wuhan Sanying Biotechnology Co., Ltd. (Wuhan, China). HRP-labeled goat anti-rat and HRP-labeled rabbit anti-goat were purchased from Kirkegaard & Perry Laboratories. BDNF (rabbit, 1:1000, No. 28205-1-AP, 28 kDa, Wuhan, China), TrkB (rabbit, 1:1000, No. 13129-1-AP, 43 kDa, Wuhan, China), β-actin (rabbit, 1:50000, No. 81115-1-RR, 42 kDa, Wuhan, China), and p-CREB (rabbit, 1:1000, No. YM8632, 92 kDa, Wuhan, China).

### 4.2. Animals and Design

Sixty male Sprague-Dawley (SD) rats (4 weeks old) were purchased from SPF Bio-technology Co., Ltd. (Beijing, China) and housed under controlled conditions (22 ± 2 °C, 50% relative humidity, 12 h light/dark cycle). All rats received standard chow and water ad libitum. The chronic depression model was established using a modified version of the widely adopted Chronic Unpredictable Mild Stress (CUMS) protocol. Rats were randomly assigned to groups using a random number table, and experimenters were blinded to treatment during behavioral assessments. The control (CON) group had free access to water and standard chow without stress stimulation, while the other five groups underwent continuous stress stimulation for 8 weeks. Stressors included: wet bedding for 24 h; 45° cage tilt for 24 h; swimming in 4 °C water for 5 min; cage shaking for 15 min; tail clipping for 5 min; fasting for 24 h; and water deprivation for 24 h [10,59,60]. The same stressor was not applied on consecutive days. After a one-week acclimation period, the rats underwent training with a 1% sucrose solution for one week. Following the sucrose preference test, and based on the 3R principle for animal experimentation and power analysis, a sample size of 10 animals per group was determined, and the rats were subsequently randomly divided into 6 groups [61]: control (CON), model (CUMS), drug (FLU), low-dose BC99 (BL), medium-dose BC99 (BM), and high-dose BC99 (BH), as shown in Figure 1. Except for the CON group, rats were housed two per cage. BC99 and fluoxetine were resuspended in 0.9% sterile saline.

The CON and CUMS groups received sterile saline via gavage, the FLU group received fluoxetine (2.1 mg/kg) [62], and the BL, BM, and BH groups received 10^6^, 10^7^, and 10^8^ CFU/g body weight of bacteria, respectively [63]. Administration continued for 8 weeks, with body weight and food intake monitored every 3 days. All behavioral tests were performed between 24 and 48 h after the final gavage. This time window was selected to exclude the acute effects of the drug. Following the completion of all behavioral experiments, the rats were fasted for 14 h and then euthanized under anesthesia, after which blood, hippocampus, colon, and cecal contents were collected for subsequent measurement and analysis of relevant indicators. The experimental design is shown in Figure 1A. This study was approved by the Ethics Committee of Henan University of Science and Technology (Protocol Number: 20250206; approval date: 21 February 2025) and carried out according to the institutional and ARRIVE guidelines.

### 4.3. Behavioral Tests

#### 4.3.1. Sucrose Preference Test (SPT)

Rats were individually housed during the experimental phase. During adaptation, two bottles of 1% (*w*/*v*) sucrose solution were placed in each cage. The following day, one sucrose bottle was replaced with plain water. Bottle positions were switched every 12 h to prevent positional bias. During testing, rats were deprived of food and water for 24 h. Two pre-weighed bottles (one with 1% sucrose solution and the other with plain water) were placed in each cage. After 4 h, both bottles were removed, and the remaining liquid volumes were measured and recorded [64].

#### 4.3.2. Tail Suspension Test (TST)

The tails of experimental rats were fixed with adhesive tape 2 cm from the tip and suspended from a horizontal bar for 6 min [65]. Cumulative immobility time during the final 4 min was recorded. Immobility was defined as the absence of struggling and complete motionlessness.

#### 4.3.3. Forced Swim Test (FST)

Individual rats were placed in a plastic cylinder filled with water (23–25 °C, 45 cm depth) for 6 min. Activities were recorded, and software was used to automatically assess immobility time. Longer immobility times indicate higher depressive behavior [66].

#### 4.3.4. Open Field Test (OFT)

The rats were acclimated in a dark, quiet room for 4 h. The OFT was conducted in an 80 cm × 80 cm open-field apparatus. Each rat was placed at the center, and the test lasted for 10 min. The apparatus was cleaned with 75% ethanol before each test. Dim lighting was used to minimize environmental influence [67]. The open-field test assessed the total locomotor activity of the rats, and the entire behavior was automatically recorded by cameras installed on the apparatus and analyzed using animal tracking software.

### 4.4. Hematoxylin and Eosin Staining (H&E)

Hippocampal and proximal colon samples were collected, rinsed with PBS, and fixed with 4% paraformaldehyde. After embedding in paraffin and fixing for 48 h, hematoxylin-eosin staining was performed. Pathological changes were observed under bright-field illumination using an optical microscope (E100, Nikon, Tokyo, Japan).

### 4.5. Measurement of Inflammatory Cytokines and BDNF by ELISA

Prepare by adding an appropriate amount of hippocampal and colonic tissue to nine times its volume of saline, and immerse in an ice-water bath (4 °C, 12,000× *g*, 15 min). Rat blood samples were centrifuged at 4 °C for 15 min at a speed of 4000 rpm to obtain plasma samples. According to the instructions provided in the kit manual, the concentrations of IL-4, IL-1β, IL-6, LPS and BDNF in the plasma were measured using an ELISA kit.

### 4.6. 16S rRNA Gene Sequencing

At the end of the 8-week modeling period, collect cecal contents, place them in sterile centrifuge tubes, quickly freeze in liquid nitrogen, and store at −80 °C to prevent DNA degradation. Total genomic DNA was extracted from samples using the cetyltrimethylammonium bromide (CTAB) method. The quality and concentration of the extracted DNA were assessed by 1% agarose gel electrophoresis, and subsequently diluted to a working concentration of 1 ng/μL with sterile water. The hypervariable V3-V4 region of the bacterial 16S rRNA gene was amplified via polymerase chain reaction (PCR) using the barcoded universal primers 341F (5′-CCTAYGGGRBGCASCAG-3′) and 806R (5′-GGACTACNNGGGTATCTAAT-3′). The PCR reactions were performed in a 15 μL mixture containing Phusion^®^ High-Fidelity PCR Master Mix (New England Biolabs, Ipswich, MA, USA), 2 μM of each forward and reverse primer, and approximately 10 ng of template DNA. The thermal cycling protocol comprised an initial denaturation at 98 °C for 1 min; followed by 30 cycles of denaturation at 98 °C for 10 s, annealing at 50 °C for 30 s, and ex-tension at 72 °C for 30 s; with a final elongation step at 72 °C for 5 min. The resulting amplicons were verified by 2% agarose gel electrophoresis, pooled in equimolar ratios, and purified. Sequencing libraries were constructed using the NEB Next^®^ Ultra DNA Library Prep Kit (New England Biolabs, Ipswich, MA, USA) following the manufacturer’s instructions, and their quality was evaluated on an Agilent 5400 system (Agilent Technologies Co., Ltd., Santa Clara, CA, USA). The qualified libraries were then sequenced on an Illumina platform to generate 250 bp paired-end reads. Sequencing service and data analysis service were provided by Wekemo Tech Group Co., Ltd. (Shenzhen, China).

### 4.7. OTU Clustering and Annotation

Paired-end reads (2 × 250 bp) generated on an Illumina NovaSeq 6000 platform (Illumina, Inc., San Diego, CA, USA). Raw data FASTQ files were imported into the format which could be operated by QIIME2 system using qiime tools import program. The demultiplexed sequences of each sample were quality filtered, trimmed, denoised, and merged, and then the qiime2 dada2 plugin was used to identify and delete chimeric sequences to obtain the feature table of OTUs (https://docs.qiime2.org/2019.1/, accessed on 24 June 2025). Any contaminating mitochondrial and chloroplast sequences were filtered using the QIIME2 feature-table plugin. Appropriate methods include ANCOM, ANOVA, Kruskal Wallis, LEfSe and DEseq2 were employed to identify the bacteria with different abundance among samples and groups [68,69]. Diversity metrics were calculated using the core-diversity plugin within QIIME2. Feature level alpha diversity indices, such as observed OTUs, Chao1 richness estimator, Shannon diversity index, and Faith’s phylogenetics diversity index were calculated to estimate the microbial diversity within an individual sample. Beta diversity distance measurements, including Bray Curtis, unweighted UniFrac and weighted UniFrac were performed to investigate the structural variation of microbial communities across samples and then visualized via principal coordinate analysis (PCoA) [70]. No additional minimum read count threshold per sample was applied for retaining OTUs, as the algorithm intrinsically handles singleton removal through its error-modeling [71]. Taxonomic assignment of OTU representative sequences was performed using the Naïve Bayes classifier in QIIME 2 against the SILVA 138.1 reference database (https://www.arb-silva.de/, accessed on 24 June 2025). The confidence threshold for taxonomic assignment was set at 0.7. Taxonomic assignment was performed from phylum to genus level (phylum, class, order, family, genus). OTUs that could not be confidently assigned to a given taxonomic level were classified as ‘unclassified’ at that level. The venn diagram was generated using EVenn (http://www.ehbio.com/test/Venn, accessed on 24 June 2025). Due to the resolution limit of 16S rRNA gene sequencing, taxonomic assignments were reported to the genus level.

### 4.8. Serum Metabolomics

UHPLC-MS/MS analysis was performed using a Vanquish UHPLC system from Microtech (Shenzhen, China) coupled to an Orbitrap Q Exactive™ HF or HF-X mass spectrometer. Separation used a Hypersil Gold column (Bremen, Germany, 100 × 2.1 mm, 1.9 μm) with a 0.2 mL/min flow rate and a 12 min gradient: 2% B (1.5 min), 2–85% B (3 min), 85–100% B (10 min), 100–2% B (10.1 min), and 2% B (12 min). The mobile phase was 0.1% formic acid in water (A) and methanol (B). MS operated in positive/negative mode with a spray voltage of 3.5 kV, capillary temperature of 320 °C, sheath gas at 35 psi, auxiliary gas at 10 L/min, S-lens RF level at 60, and auxiliary gas heater at 350 °C. We annotated these metabolites by searching against the KEGG database (https://www.genome.jp/kegg/pathway.html, accessed on 24 June 2025), HMDB database (https://hmdb.ca/metabolites, accessed on 24 June 2025), and LIPIDMaps database (http://www.lipidmaps.org/, accessed on 24 June 2025).

### 4.9. Short-Chain Fatty Acid (SCFA) Analysis

A total of 0.20 g of fresh fecal sample was weighed and mixed with 1.60 mL of sterile deionized water. After thorough vortexing, the mixture was allowed to stand at room temperature for 20 min. It was then centrifuged at 4 °C and 15,000 rpm for 15 min. The supernatant was transferred to a new EP tube. Another 1.60 mL of sterile deionized water was added to the fecal pellet, and the above steps were repeated. The supernatants from both extractions were combined, filtered through a 0.22 μm membrane, and 0.20 mL of the filtrate was taken for gas chromatography (GC) analysis [32].

For GC sample preparation, 0.20 mL of the filtered supernatant was mixed with 0.70 mL of sterile water and 0.10 mL of n-butanol solution (100 μg/mL). The standard solution was prepared using six short-chain fatty acid (SCFA) standards: acetic acid, propionic acid, isobutyric acid, butyric acid, isovaleric acid, and valeric acid. To construct the calibration curve, 0.20 mL of the mixed standard solution was combined with 0.70 mL of sterile water and 0.10 mL of n-butanol, yielding a final n-butanol concentration of 100 μg/mL. n-butanol was used as an internal reference, and the average peak area from multiple injections was applied to correct for inter-batch variation. The standard curve was generated by plotting the concentration of each SCFA standard against the corresponding peak area.

The gas chromatography conditions were as follows: a JN 5MS capillary column (Hailong Instrument Factory, Shanghai, China; 30 m × 0.25 mm, 0.25 μm); inlet temperature 250 °C; injection volume 1 μL. The temperature program was set as follows: initial temperature 40 °C held for 1 min; increased at 8 °C/min to 60 °C and held for 1 min; then raised at 10 °C/min to 70 °C and held for 1 min; finally it was increased at 20 °C/min to 220 °C and held for 10 min. The carrier gas flow was maintained at a constant rate of 1.5 mL/min in splitless mode.

### 4.10. Sodium Butyrate Verification Experiment

The sodium butyrate validation experiment was conducted in parallel and independently from the main CUMS experiment, using the same rat strain, CUMS modeling procedure, and 8-week intervention period. Thirty rats were randomly divided into control, model, and sodium butyrate groups, with 10 rats per group. The intervention methods were the same as described above. The dose for the sodium butyrate validation group was 30 mg/kg body weight [37]. At the end of the experiment, colonic H&E sections were observed, and BDNF, LPS, and inflammatory factors (IL-4, IL-6, IL-8) were determined as described above, using services from Shanghai Hepeng Biotechnology Co., Ltd. (Shanghai, China).

### 4.11. Western Blot Analysis

Hippocampal tissues were lysed in RIPA buffer containing protease and phosphatase inhibitors. Proteins were separated by SDS-PAGE and transferred to PVDF membranes. Membranes were incubated with primary antibodies against BDNF, TrkB, p-CREB, and β-actin, followed by HRP-conjugated secondary antibodies. Band densities of each sample were quantified using ImageJ software (1.53a) and expressed as the ratio of the target protein signal to that of the β-actin loading control. All Western blot experiments were independently repeated three times, and the results are presented as mean ± SD. Intergroup comparisons were performed using one-way analysis of variance (ANOVA) followed by Tukey’s post hoc test.

### 4.12. Statistical Analysis

The charts were created using GraphPad Prism 10.0 software. Statistical analyses were performed using SPSS 25.0 software. Experimental data are presented as mean ± standard deviation (n = 6) and 95% confidence intervals. For comparisons between groups: if the data meet the assumptions of normality and homogeneity of variance, an independent samples *t*-test can be used. If these assumptions are not met, the Mann–Whitney U test can be used. For multiple group comparisons: if the data meet the assumptions of normality and homogeneity of variance, one-way analysis of variance (ANOVA) can be used, and Tukey HSD post hoc multiple comparison test. If the data do not meet the assumptions for ANOVA, the Kruskal–Wallis test can be applied; subsequently, a Dunn’s post hoc multiple comparison test was performed (with Bonferroni correction). All result figure legends clearly indicate the sample size for each group. With * *p* < 0.05 indicating a difference, ** *p* < 0.01 indicating a significant difference, *** *p* < 0.001 indicating a highly significant difference, and ns indicating for no statistical difference.

## 5. Conclusions

This study demonstrates an association between butyrate and the antidepressant-like effects of *W. coagulans* BC99, and shows that butyrate alone recapitulates several of these effects, without establishing a causal mediating role, by demonstrating that butyrate supplementation alone recapitulates several probiotic-induced behavioral, anti-inflammatory, and neurotrophic benefits including activation of the BDNF/TrkB/CREB signaling pathway. Our findings are consistent with the hypothesis that microbial metabolite production may influence host neuroimmune outcomes. In summary, our findings are consistent with the hypothesis that butyrate may serve as a functional mediator of the antidepressant effects of *Weizmannia coagulans* BC99. This study provides a conceptual framework for understanding the mechanisms underlying probiotic action. Furthermore, our results suggest that butyrate-enhancing strategies warrant further investigation as a potential nutritional intervention for depression.

## Data Availability

The raw 16S rRNA gene sequencing data have been deposited in the NCBI Sequence Read Archive (SRA) under BioProject accession number PRJNA1458870. Due to an ongoing investigation, the data are under embargo until 1 May 2027, and will be made publicly available at that time. During the embargo period, data are available from the corresponding author upon reasonable request.

## Supplementary Materials

The following supporting information can be downloaded at: https://www.mdpi.com/article/10.3390/ijms27094082/s1.

## Abbreviations

CUMS	Chronic unpredictable mild stress
GABA	Gamma-aminobutyric acid
LPS	Lipopolysaccharide
BC99	*Weizmannia coagulans* BC99
BDNF	Brain-derived neurotrophic factor
