# Supplementary figures and images for: Butyrate Is Associated with the Antidepressant Effects of Weizmannia coagulans BC99: Functional Similarity of a Microbial Metabolite in the Microbiota–Gut–Brain Axis
Source: Int J Mol Sci. 2026 May 2;27(9):4082. doi: 10.3390/ijms27094082 (PMC13163339; doi:10.3390/ijms27094082)

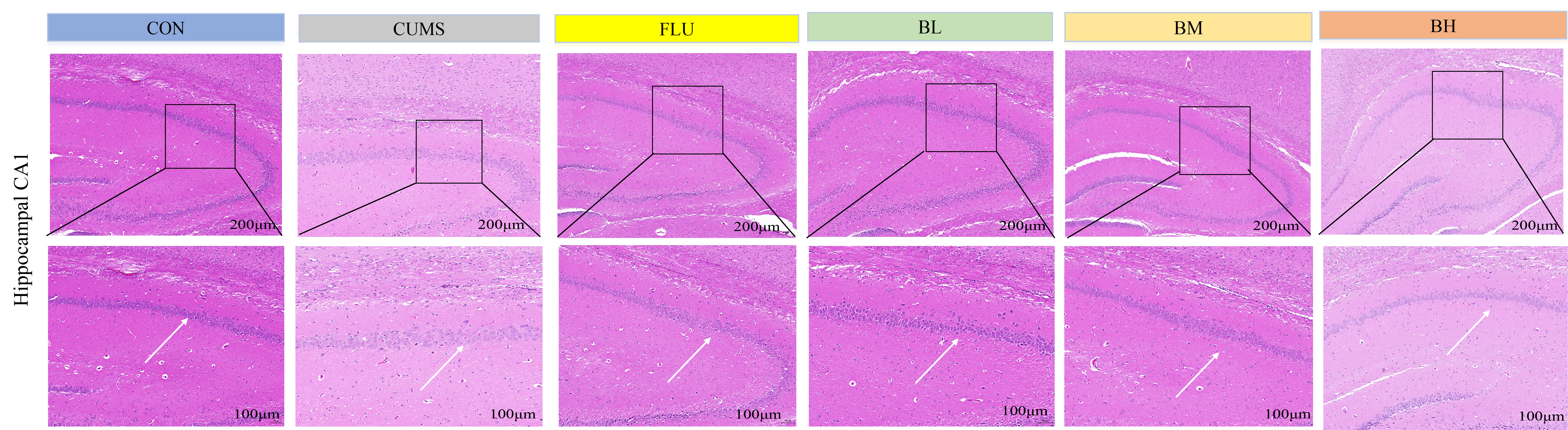

Supplement: Supplementary file 1 [file ijms-27-04082-s001.zip › Figure.S1 Effect of BC99 Intervention on the Hippocampus of CUMS Rats.tif]

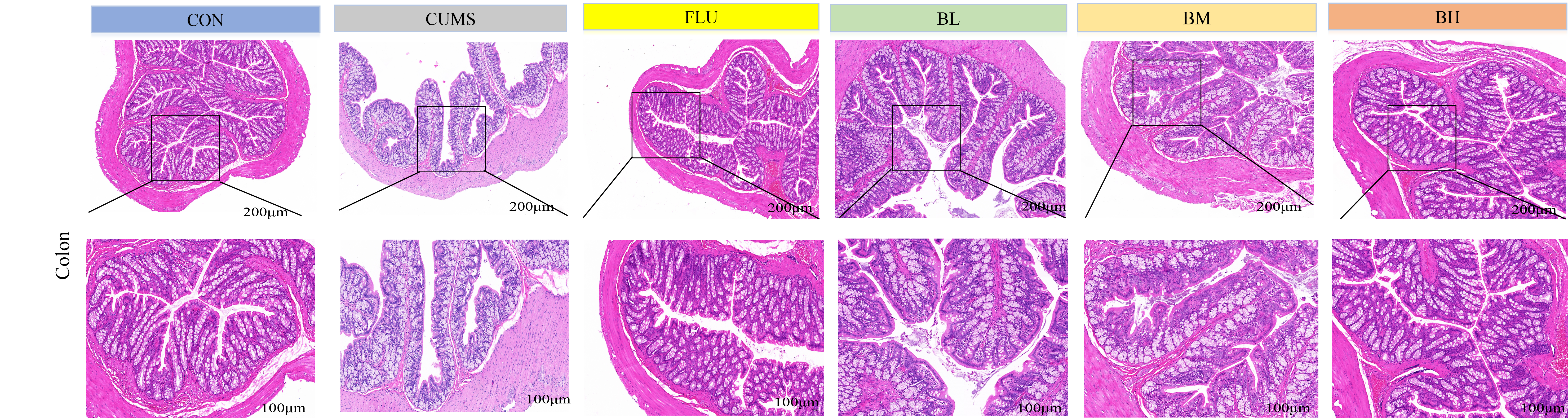

Supplement: Supplementary file 1 [file ijms-27-04082-s001.zip › Figure.S2 Effect of BC99 Intervention on the Colon of CUMS Rats..tif]

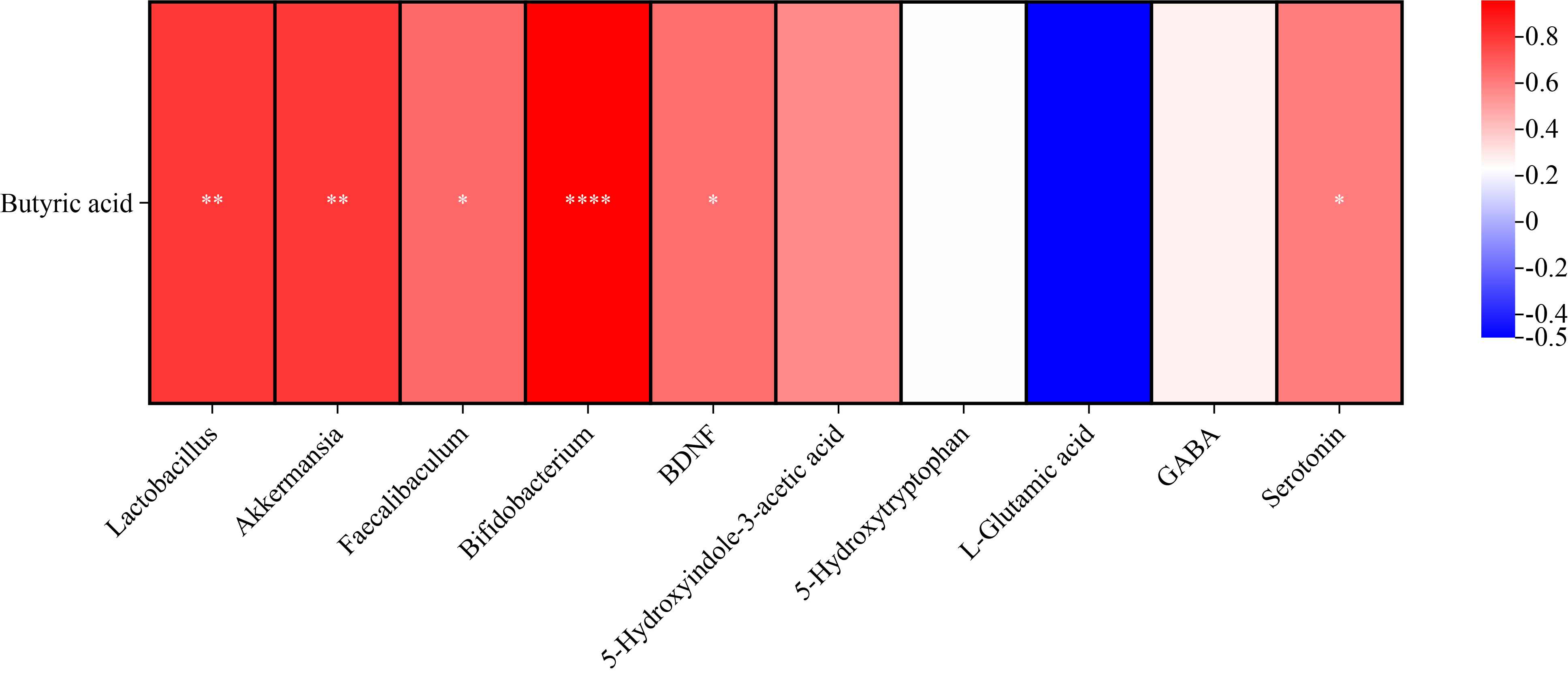

Supplement: Supplementary file 1 [file ijms-27-04082-s001.zip › Figure.S3 Analysis of the correlation between butyric acid and different strains, behavior, and BDNF.tif]

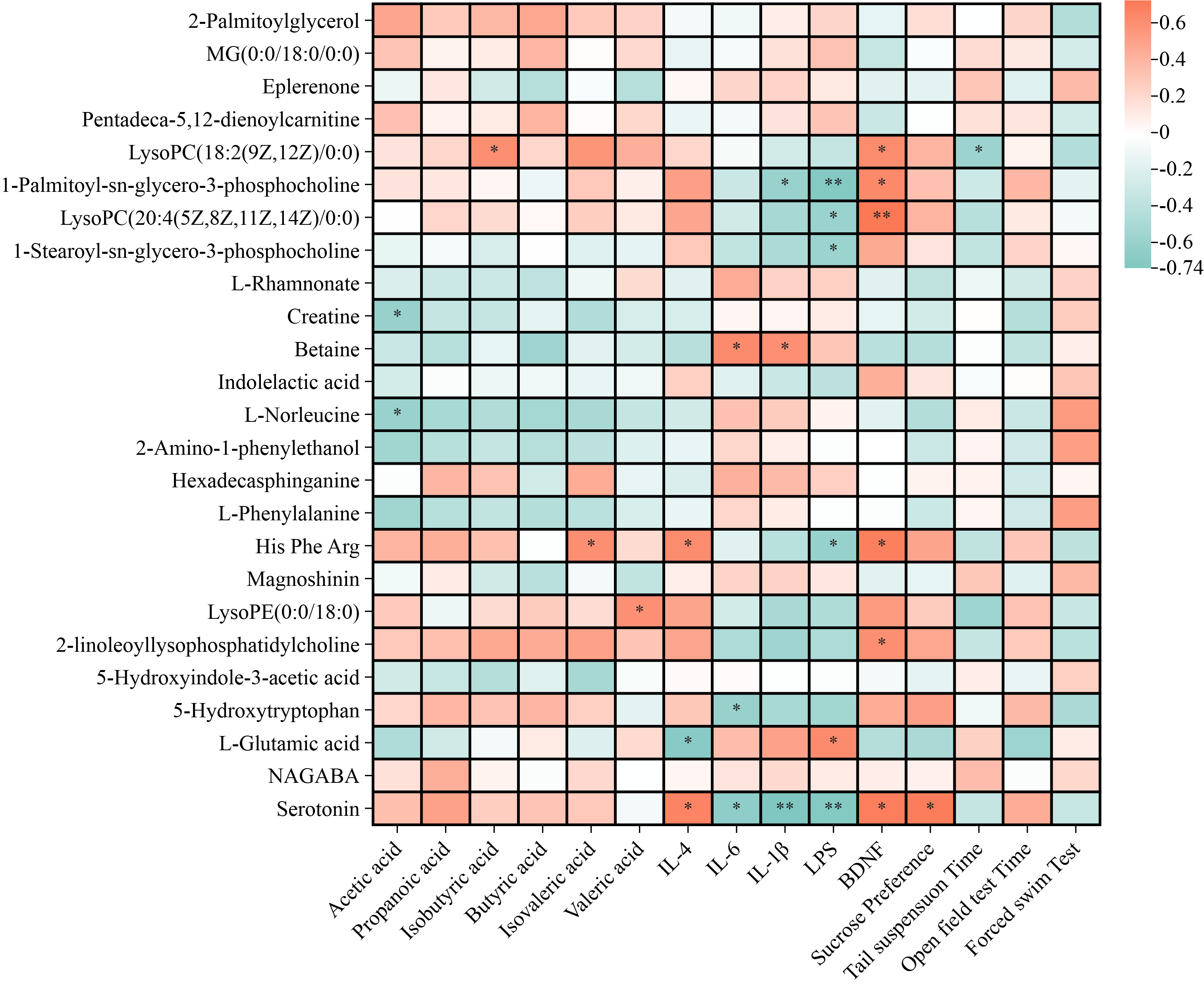

Supplement: Supplementary file 1 [file ijms-27-04082-s001.zip › Figure.S4 Correlation analysis of differential metabolites with biochemical indicators, SCFAs, and behavior.tif]

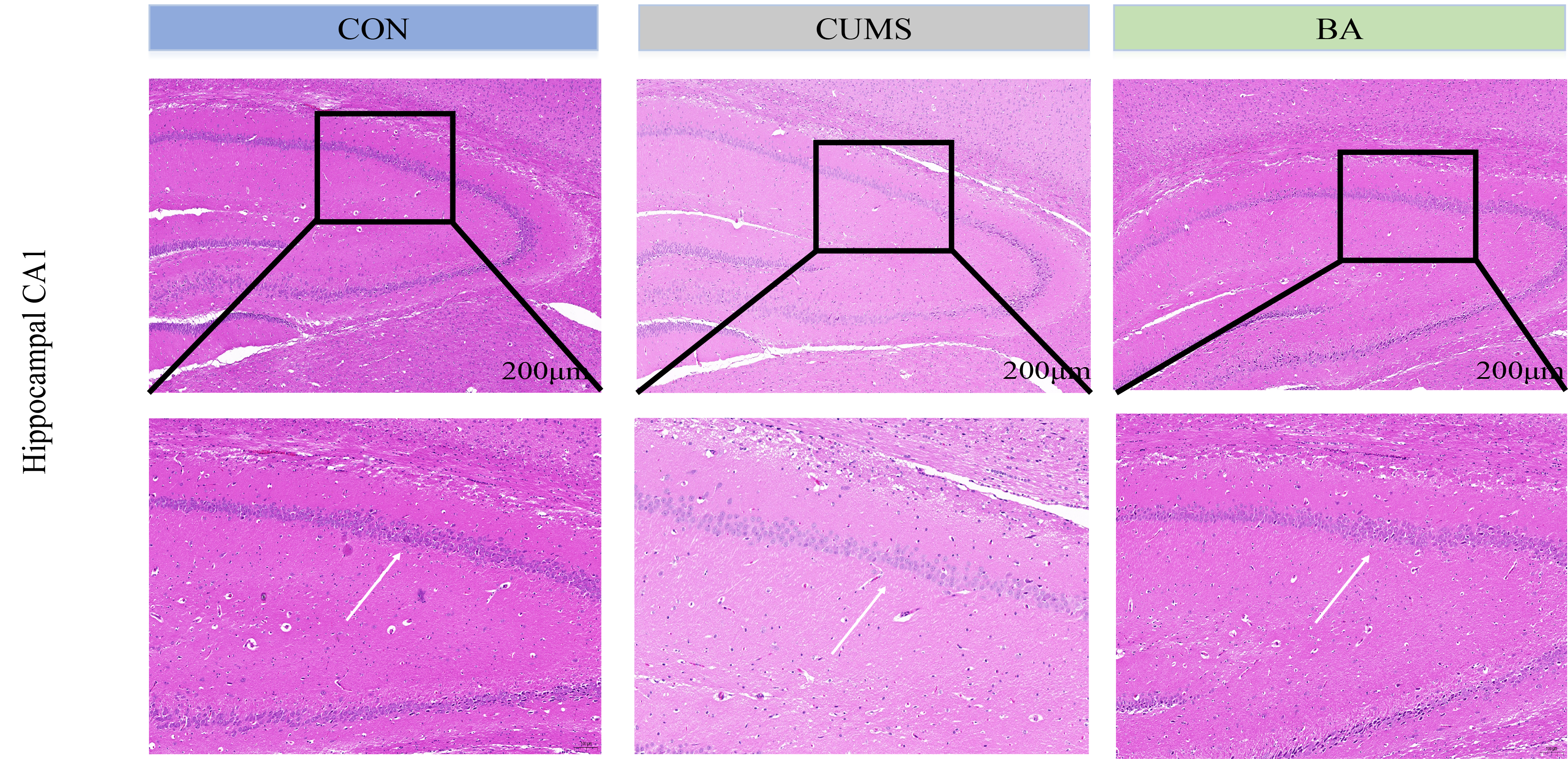

Supplement: Supplementary file 1 [file ijms-27-04082-s001.zip › Figure.S5 Effect of BA Intervention on the Hippocampus of CUMS Rats..tif]

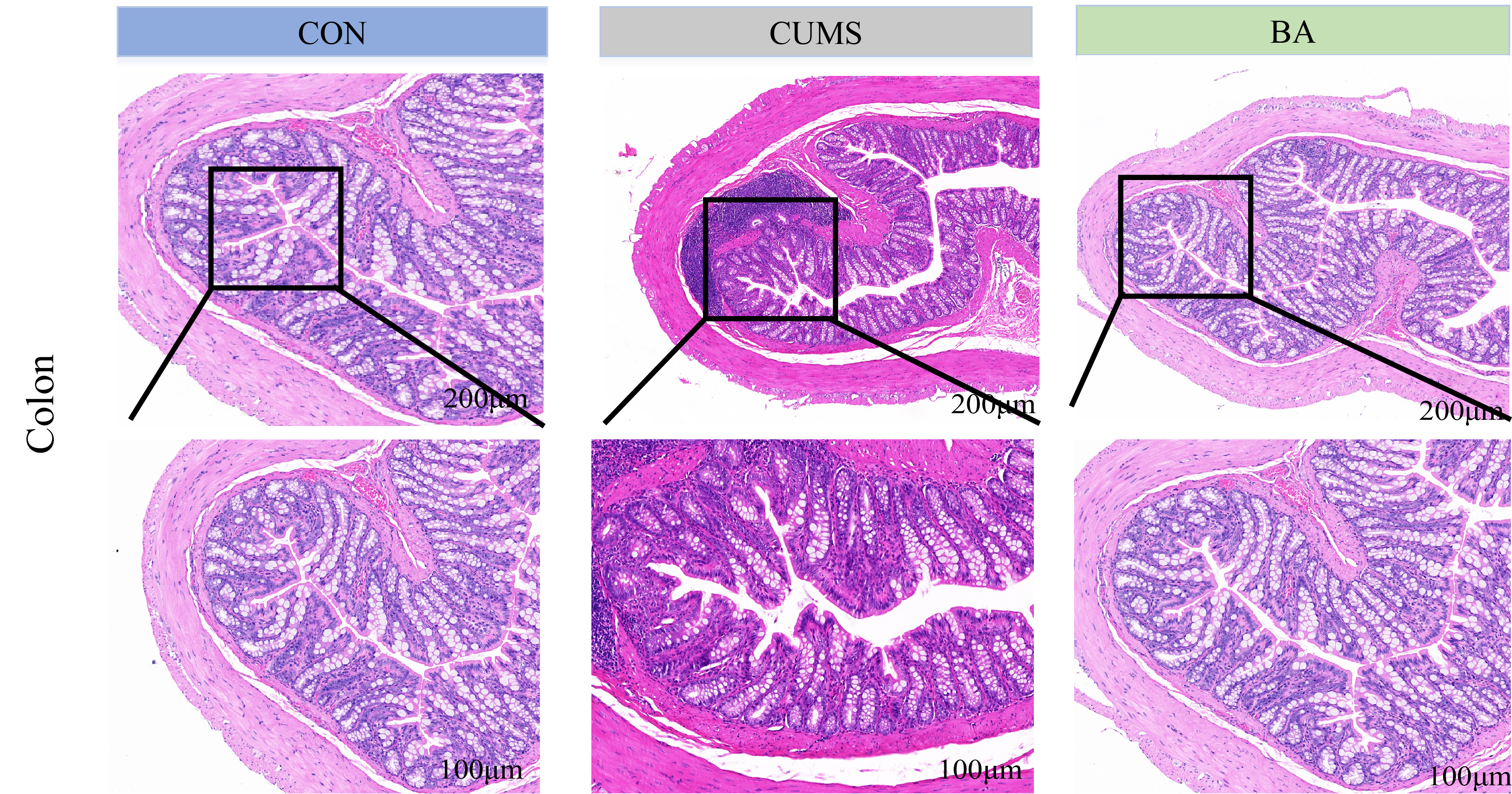

Supplement: Supplementary file 1 [file ijms-27-04082-s001.zip › Figure.S6 Effect of BC99 Intervention on the Colon of CUMS Rats..tif]
